# Supplementary material for: Modeling glioblastoma heterogeneity as a dynamic network of cell states
Source: Mol Syst Biol. 2021 Sep 16;17(9):e10105. doi: 10.15252/msb.202010105 (PMC8444284; doi:10.15252/msb.202010105)
Supplement: Supplementary file 6 — Source Data for Figure 5 [file MSB-17-e10105-s004.zip › Figure5A_sourcedata/GSEA_3071/nosigdb_stateI.GseaPreranked.1622795145664/FELDMAN_2020_S-G2.html]

Details for gene set FELDMAN\_2020\_S-G2[GSEA]

|  || Dataset | state13071 |
| Phenotype | NoPhenotypeAvailable |
| Upregulated in class | na\_neg |
| GeneSet | FELDMAN\_2020\_S-G2 |
| Enrichment Score (ES) | -0.58649313 |
| Normalized Enrichment Score (NES) | -3.5508957 |
| Nominal p-value | 0.0 |
| FDR q-value | 0.0 |
| FWER p-Value | 0.0 |
Table: GSEA Results Summary

  

Fig 1: Enrichment plot: FELDMAN\_2020\_S-G2      
 Profile of the Running ES Score & Positions of GeneSet Members on the Rank Ordered List

  

| PROBE | GENE SYMBOL | GENE\_TITLE | RANK IN GENE LIST | RANK METRIC SCORE | RUNNING ES | CORE ENRICHMENT || 1 | MIAT |  |  | 41 | 1.422 | -0.0046 | No |
| 2 | SERPINF1 |  |  | 71 | 1.211 | -0.0067 | No |
| 3 | ID3 |  |  | 86 | 1.152 | -0.0046 | No |
| 4 | SPARC |  |  | 106 | 1.067 | -0.0044 | No |
| 5 | CTGF |  |  | 152 | 0.913 | -0.0131 | No |
| 6 | IGFBP5 |  |  | 198 | 0.833 | -0.0223 | No |
| 7 | IFITM3 |  |  | 268 | 0.714 | -0.0395 | No |
| 8 | MAGED2 |  |  | 302 | 0.669 | -0.0459 | No |
| 9 | RGCC |  |  | 432 | 0.566 | -0.0825 | No |
| 10 | PRDX1 |  |  | 447 | 0.549 | -0.0837 | No |
| 11 | LAPTM4A |  |  | 553 | 0.478 | -0.1133 | No |
| 12 | FTL |  |  | 564 | 0.473 | -0.1138 | No |
| 13 | KLF6 |  |  | 647 | 0.433 | -0.1366 | No |
| 14 | TMEM59 |  |  | 665 | 0.424 | -0.1394 | No |
| 15 | BAMBI |  |  | 740 | 0.400 | -0.1600 | No |
| 16 | ARL6IP5 |  |  | 756 | 0.395 | -0.1623 | No |
| 17 | POLD4 |  |  | 810 | 0.378 | -0.1765 | No |
| 18 | DDIT4 |  |  | 865 | 0.361 | -0.1911 | No |
| 19 | PLD3 |  |  | 980 | 0.331 | -0.2244 | No |
| 20 | RABAC1 |  |  | 1092 | 0.308 | -0.2568 | No |
| 21 | MALAT1 |  |  | 1252 | 0.276 | -0.3043 | No |
| 22 | PHLDA1 |  |  | 1262 | 0.275 | -0.3055 | No |
| 23 | CCDC34 |  |  | 1376 | 0.255 | -0.3388 | No |
| 24 | ARL6IP6 |  |  | 1577 | -0.265 | -0.3989 | No |
| 25 | SUPT16H |  |  | 1588 | -0.266 | -0.4005 | No |
| 26 | HSPA8 |  |  | 1605 | -0.268 | -0.4039 | No |
| 27 | HNRNPF |  |  | 1636 | -0.272 | -0.4116 | No |
| 28 | SAE1 |  |  | 1638 | -0.273 | -0.4104 | No |
| 29 | SMC3 |  |  | 1650 | -0.274 | -0.4123 | No |
| 30 | BUB3 |  |  | 1687 | -0.278 | -0.4218 | No |
| 31 | NXT1 |  |  | 1722 | -0.283 | -0.4307 | No |
| 32 | PSMC3 |  |  | 1752 | -0.287 | -0.4380 | No |
| 33 | CALM2 |  |  | 1763 | -0.289 | -0.4394 | No |
| 34 | MIS18BP1 |  |  | 1764 | -0.289 | -0.4378 | No |
| 35 | CHCHD2 |  |  | 1822 | -0.296 | -0.4537 | No |
| 36 | RAN |  |  | 1907 | -0.306 | -0.4778 | No |
| 37 | KPNA2 |  |  | 1944 | -0.312 | -0.4872 | No |
| 38 | SPDL1 |  |  | 1968 | -0.314 | -0.4925 | No |
| 39 | SNRPA1 |  |  | 1984 | -0.316 | -0.4953 | No |
| 40 | CEP78 |  |  | 2000 | -0.317 | -0.4982 | No |
| 41 | ACYP1 |  |  | 2052 | -0.326 | -0.5120 | No |
| 42 | SNRPB |  |  | 2064 | -0.328 | -0.5136 | No |
| 43 | CDKN1A |  |  | 2083 | -0.332 | -0.5173 | No |
| 44 | RRS1 |  |  | 2091 | -0.333 | -0.5175 | No |
| 45 | EIF1AX |  |  | 2103 | -0.335 | -0.5190 | No |
| 46 | BRIX1 |  |  | 2135 | -0.340 | -0.5267 | No |
| 47 | HNRNPDL |  |  | 2142 | -0.342 | -0.5266 | No |
| 48 | HSP90AA1 |  |  | 2156 | -0.344 | -0.5287 | No |
| 49 | COPS3 |  |  | 2175 | -0.348 | -0.5323 | No |
| 50 | MXD3 |  |  | 2201 | -0.352 | -0.5380 | No |
| 51 | CENPA |  |  | 2220 | -0.357 | -0.5415 | No |
| 52 | SMC1A |  |  | 2240 | -0.361 | -0.5453 | No |
| 53 | SNRPE |  |  | 2247 | -0.362 | -0.5451 | No |
| 54 | HMGN1 |  |  | 2248 | -0.363 | -0.5431 | No |
| 55 | TUBB4B |  |  | 2291 | -0.370 | -0.5539 | No |
| 56 | SCD5 |  |  | 2299 | -0.373 | -0.5540 | No |
| 57 | DONSON |  |  | 2316 | -0.375 | -0.5568 | No |
| 58 | CKS1B |  |  | 2332 | -0.380 | -0.5593 | No |
| 59 | CKAP2 |  |  | 2357 | -0.385 | -0.5645 | No |
| 60 | ZWILCH |  |  | 2358 | -0.385 | -0.5624 | No |
| 61 | PSIP1 |  |  | 2364 | -0.386 | -0.5617 | No |
| 62 | HSPD1 |  |  | 2396 | -0.392 | -0.5691 | No |
| 63 | CENPW |  |  | 2402 | -0.393 | -0.5684 | No |
| 64 | DCBLD2 |  |  | 2407 | -0.393 | -0.5674 | No |
| 65 | WDR34 |  |  | 2414 | -0.395 | -0.5670 | No |
| 66 | HAUS1 |  |  | 2423 | -0.397 | -0.5673 | No |
| 67 | H2AFV |  |  | 2431 | -0.398 | -0.5672 | No |
| 68 | HPRT1 |  |  | 2454 | -0.402 | -0.5717 | No |
| 69 | SNRPG |  |  | 2459 | -0.404 | -0.5706 | No |
| 70 | CKS2 |  |  | 2466 | -0.404 | -0.5702 | No |
| 71 | PDCD5 |  |  | 2476 | -0.406 | -0.5707 | No |
| 72 | PLAT |  |  | 2501 | -0.415 | -0.5758 | No |
| 73 | CACYBP |  |  | 2536 | -0.424 | -0.5838 | No |
| 74 | PARP2 |  |  | 2542 | -0.425 | -0.5830 | No |
| 75 | PAICS |  |  | 2545 | -0.426 | -0.5812 | No |
| 76 | TEX30 |  |  | 2556 | -0.428 | -0.5819 | No |
| 77 | H2AFZ |  |  | 2572 | -0.433 | -0.5841 | Yes |
| 78 | RPL22L1 |  |  | 2576 | -0.434 | -0.5825 | Yes |
| 79 | LRR1 |  |  | 2579 | -0.434 | -0.5807 | Yes |
| 80 | SSRP1 |  |  | 2580 | -0.434 | -0.5783 | Yes |
| 81 | BEX4 |  |  | 2584 | -0.435 | -0.5767 | Yes |
| 82 | IER2 |  |  | 2587 | -0.436 | -0.5749 | Yes |
| 83 | TMEM106C |  |  | 2592 | -0.437 | -0.5737 | Yes |
| 84 | ANP32B |  |  | 2593 | -0.438 | -0.5712 | Yes |
| 85 | C12orf75 |  |  | 2607 | -0.441 | -0.5727 | Yes |
| 86 | GGCT |  |  | 2610 | -0.442 | -0.5708 | Yes |
| 87 | SRSF7 |  |  | 2619 | -0.445 | -0.5708 | Yes |
| 88 | SRSF3 |  |  | 2631 | -0.447 | -0.5717 | Yes |
| 89 | HIRIP3 |  |  | 2635 | -0.449 | -0.5701 | Yes |
| 90 | DCK |  |  | 2636 | -0.449 | -0.5675 | Yes |
| 91 | EIF2S1 |  |  | 2638 | -0.450 | -0.5653 | Yes |
| 92 | DUT |  |  | 2640 | -0.450 | -0.5631 | Yes |
| 93 | RNPS1 |  |  | 2641 | -0.450 | -0.5606 | Yes |
| 94 | DCTPP1 |  |  | 2644 | -0.451 | -0.5586 | Yes |
| 95 | UBE2I |  |  | 2648 | -0.452 | -0.5570 | Yes |
| 96 | SET |  |  | 2652 | -0.455 | -0.5554 | Yes |
| 97 | EXOSC8 |  |  | 2659 | -0.456 | -0.5547 | Yes |
| 98 | CCNA2 |  |  | 2667 | -0.462 | -0.5542 | Yes |
| 99 | PGP |  |  | 2681 | -0.468 | -0.5556 | Yes |
| 100 | MND1 |  |  | 2682 | -0.468 | -0.5529 | Yes |
| 101 | KIF22 |  |  | 2683 | -0.468 | -0.5503 | Yes |
| 102 | CMC2 |  |  | 2694 | -0.470 | -0.5507 | Yes |
| 103 | SNRPD1 |  |  | 2701 | -0.471 | -0.5499 | Yes |
| 104 | ANLN |  |  | 2702 | -0.472 | -0.5473 | Yes |
| 105 | HAT1 |  |  | 2703 | -0.472 | -0.5446 | Yes |
| 106 | PRC1 |  |  | 2705 | -0.472 | -0.5423 | Yes |
| 107 | CENPN |  |  | 2708 | -0.474 | -0.5402 | Yes |
| 108 | MELK |  |  | 2709 | -0.474 | -0.5375 | Yes |
| 109 | TUBG1 |  |  | 2715 | -0.475 | -0.5364 | Yes |
| 110 | NDC80 |  |  | 2723 | -0.478 | -0.5359 | Yes |
| 111 | YWHAH |  |  | 2725 | -0.479 | -0.5335 | Yes |
| 112 | CYCS |  |  | 2727 | -0.479 | -0.5311 | Yes |
| 113 | RPA3 |  |  | 2730 | -0.480 | -0.5290 | Yes |
| 114 | SHCBP1 |  |  | 2733 | -0.480 | -0.5269 | Yes |
| 115 | CHCHD3 |  |  | 2749 | -0.485 | -0.5288 | Yes |
| 116 | CENPF |  |  | 2761 | -0.490 | -0.5294 | Yes |
| 117 | SLC25A5 |  |  | 2765 | -0.493 | -0.5276 | Yes |
| 118 | TUBA1A |  |  | 2771 | -0.495 | -0.5263 | Yes |
| 119 | BRCA2 |  |  | 2772 | -0.495 | -0.5235 | Yes |
| 120 | ENC1 |  |  | 2776 | -0.496 | -0.5217 | Yes |
| 121 | NUF2 |  |  | 2781 | -0.497 | -0.5201 | Yes |
| 122 | SRSF2 |  |  | 2782 | -0.497 | -0.5173 | Yes |
| 123 | TUBA1B |  |  | 2785 | -0.498 | -0.5151 | Yes |
| 124 | DTYMK |  |  | 2798 | -0.501 | -0.5160 | Yes |
| 125 | FBL |  |  | 2804 | -0.502 | -0.5147 | Yes |
| 126 | HMGB1 |  |  | 2806 | -0.503 | -0.5122 | Yes |
| 127 | HNRNPAB |  |  | 2816 | -0.508 | -0.5121 | Yes |
| 128 | SIVA1 |  |  | 2822 | -0.510 | -0.5108 | Yes |
| 129 | ASF1B |  |  | 2824 | -0.510 | -0.5082 | Yes |
| 130 | CDCA3 |  |  | 2829 | -0.513 | -0.5066 | Yes |
| 131 | CDKN2D |  |  | 2832 | -0.514 | -0.5043 | Yes |
| 132 | TOP2A |  |  | 2842 | -0.517 | -0.5041 | Yes |
| 133 | TOMM40 |  |  | 2845 | -0.519 | -0.5018 | Yes |
| 134 | CEP55 |  |  | 2852 | -0.522 | -0.5007 | Yes |
| 135 | CKAP2L |  |  | 2857 | -0.523 | -0.4990 | Yes |
| 136 | NUDT1 |  |  | 2859 | -0.524 | -0.4964 | Yes |
| 137 | RFC5 |  |  | 2862 | -0.526 | -0.4940 | Yes |
| 138 | TRIP13 |  |  | 2865 | -0.526 | -0.4917 | Yes |
| 139 | HJURP |  |  | 2869 | -0.527 | -0.4897 | Yes |
| 140 | POLR3K |  |  | 2872 | -0.528 | -0.4873 | Yes |
| 141 | PTTG1 |  |  | 2895 | -0.540 | -0.4910 | Yes |
| 142 | TMEM158 |  |  | 2900 | -0.543 | -0.4892 | Yes |
| 143 | RAD23A |  |  | 2914 | -0.550 | -0.4901 | Yes |
| 144 | PRCP |  |  | 2919 | -0.553 | -0.4882 | Yes |
| 145 | NETO2 |  |  | 2920 | -0.556 | -0.4851 | Yes |
| 146 | ARHGAP11A |  |  | 2921 | -0.556 | -0.4820 | Yes |
| 147 | CMSS1 |  |  | 2931 | -0.561 | -0.4816 | Yes |
| 148 | TRA2B |  |  | 2933 | -0.563 | -0.4787 | Yes |
| 149 | ATAD5 |  |  | 2935 | -0.563 | -0.4759 | Yes |
| 150 | PIN1 |  |  | 2936 | -0.565 | -0.4727 | Yes |
| 151 | CENPH |  |  | 2945 | -0.569 | -0.4719 | Yes |
| 152 | MIS18A |  |  | 2947 | -0.570 | -0.4690 | Yes |
| 153 | DSN1 |  |  | 2949 | -0.571 | -0.4661 | Yes |
| 154 | KIF4A |  |  | 2953 | -0.574 | -0.4638 | Yes |
| 155 | TACC3 |  |  | 2956 | -0.575 | -0.4612 | Yes |
| 156 | KIF23 |  |  | 2961 | -0.579 | -0.4592 | Yes |
| 157 | MZT1 |  |  | 2964 | -0.580 | -0.4565 | Yes |
| 158 | ZFAND6 |  |  | 2966 | -0.581 | -0.4536 | Yes |
| 159 | GGH |  |  | 2975 | -0.585 | -0.4527 | Yes |
| 160 | CTNNAL1 |  |  | 2976 | -0.585 | -0.4494 | Yes |
| 161 | POP7 |  |  | 2980 | -0.588 | -0.4470 | Yes |
| 162 | VRK1 |  |  | 2983 | -0.592 | -0.4443 | Yes |
| 163 | SMC2 |  |  | 2984 | -0.592 | -0.4410 | Yes |
| 164 | SKA3 |  |  | 2985 | -0.592 | -0.4377 | Yes |
| 165 | FOSL1 |  |  | 3003 | -0.604 | -0.4395 | Yes |
| 166 | TPX2 |  |  | 3006 | -0.606 | -0.4367 | Yes |
| 167 | C19orf48 |  |  | 3013 | -0.608 | -0.4351 | Yes |
| 168 | SNRNP25 |  |  | 3018 | -0.613 | -0.4329 | Yes |
| 169 | TCF19 |  |  | 3019 | -0.613 | -0.4295 | Yes |
| 170 | RTKN2 |  |  | 3020 | -0.613 | -0.4260 | Yes |
| 171 | TFDP1 |  |  | 3027 | -0.617 | -0.4244 | Yes |
| 172 | DNMT1 |  |  | 3035 | -0.622 | -0.4230 | Yes |
| 173 | PARP1 |  |  | 3039 | -0.626 | -0.4204 | Yes |
| 174 | RBBP8 |  |  | 3053 | -0.637 | -0.4208 | Yes |
| 175 | AURKB |  |  | 3056 | -0.639 | -0.4179 | Yes |
| 176 | SAC3D1 |  |  | 3073 | -0.655 | -0.4191 | Yes |
| 177 | MAP3K7CL |  |  | 3076 | -0.657 | -0.4160 | Yes |
| 178 | CDC45 |  |  | 3081 | -0.661 | -0.4135 | Yes |
| 179 | GTSE1 |  |  | 3083 | -0.663 | -0.4101 | Yes |
| 180 | CDCA4 |  |  | 3084 | -0.663 | -0.4064 | Yes |
| 181 | TMPO |  |  | 3088 | -0.666 | -0.4035 | Yes |
| 182 | MAD2L1 |  |  | 3099 | -0.675 | -0.4028 | Yes |
| 183 | NASP |  |  | 3106 | -0.682 | -0.4008 | Yes |
| 184 | KIFC1 |  |  | 3110 | -0.684 | -0.3979 | Yes |
| 185 | LSM4 |  |  | 3115 | -0.688 | -0.3953 | Yes |
| 186 | PA2G4 |  |  | 3116 | -0.690 | -0.3914 | Yes |
| 187 | DBF4 |  |  | 3117 | -0.690 | -0.3875 | Yes |
| 188 | LMNB1 |  |  | 3121 | -0.692 | -0.3845 | Yes |
| 189 | SMC4 |  |  | 3126 | -0.699 | -0.3818 | Yes |
| 190 | CENPK |  |  | 3127 | -0.700 | -0.3779 | Yes |
| 191 | RFC4 |  |  | 3129 | -0.701 | -0.3742 | Yes |
| 192 | HIST1H2AH |  |  | 3139 | -0.716 | -0.3730 | Yes |
| 193 | GMNN |  |  | 3140 | -0.719 | -0.3689 | Yes |
| 194 | BTG3 |  |  | 3148 | -0.727 | -0.3670 | Yes |
| 195 | CDK1 |  |  | 3149 | -0.727 | -0.3629 | Yes |
| 196 | HNRNPD |  |  | 3157 | -0.738 | -0.3609 | Yes |
| 197 | MKI67 |  |  | 3158 | -0.738 | -0.3567 | Yes |
| 198 | ORC6 |  |  | 3159 | -0.738 | -0.3526 | Yes |
| 199 | TK1 |  |  | 3162 | -0.745 | -0.3490 | Yes |
| 200 | RNASEH2B |  |  | 3166 | -0.747 | -0.3457 | Yes |
| 201 | DNAJC9 |  |  | 3167 | -0.747 | -0.3415 | Yes |
| 202 | STMN1 |  |  | 3171 | -0.751 | -0.3382 | Yes |
| 203 | MCM3 |  |  | 3182 | -0.761 | -0.3370 | Yes |
| 204 | USP1 |  |  | 3183 | -0.762 | -0.3327 | Yes |
| 205 | CDCA2 |  |  | 3189 | -0.767 | -0.3299 | Yes |
| 206 | PSMC3IP |  |  | 3194 | -0.772 | -0.3268 | Yes |
| 207 | PXMP2 |  |  | 3200 | -0.782 | -0.3240 | Yes |
| 208 | BIRC5 |  |  | 3208 | -0.793 | -0.3217 | Yes |
| 209 | FBXO5 |  |  | 3212 | -0.797 | -0.3181 | Yes |
| 210 | DIAPH3 |  |  | 3213 | -0.800 | -0.3136 | Yes |
| 211 | CARHSP1 |  |  | 3214 | -0.801 | -0.3091 | Yes |
| 212 | RRM1 |  |  | 3215 | -0.801 | -0.3046 | Yes |
| 213 | SPC25 |  |  | 3219 | -0.806 | -0.3010 | Yes |
| 214 | UBE2C |  |  | 3225 | -0.812 | -0.2979 | Yes |
| 215 | SYNE2 |  |  | 3231 | -0.820 | -0.2948 | Yes |
| 216 | NUSAP1 |  |  | 3237 | -0.825 | -0.2917 | Yes |
| 217 | HMGN2 |  |  | 3242 | -0.833 | -0.2883 | Yes |
| 218 | RFC2 |  |  | 3245 | -0.839 | -0.2842 | Yes |
| 219 | CENPM |  |  | 3269 | -0.881 | -0.2863 | Yes |
| 220 | SOX9 |  |  | 3271 | -0.883 | -0.2816 | Yes |
| 221 | FEN1 |  |  | 3278 | -0.890 | -0.2785 | Yes |
| 222 | H2AFX |  |  | 3282 | -0.898 | -0.2743 | Yes |
| 223 | HELLS |  |  | 3284 | -0.901 | -0.2696 | Yes |
| 224 | RANBP1 |  |  | 3290 | -0.911 | -0.2660 | Yes |
| 225 | ZWINT |  |  | 3296 | -0.925 | -0.2623 | Yes |
| 226 | TM7SF2 |  |  | 3298 | -0.929 | -0.2574 | Yes |
| 227 | HMGA1 |  |  | 3299 | -0.931 | -0.2521 | Yes |
| 228 | PCNA |  |  | 3319 | -0.963 | -0.2526 | Yes |
| 229 | GPM6A |  |  | 3328 | -0.976 | -0.2495 | Yes |
| 230 | UBE2T |  |  | 3330 | -0.979 | -0.2443 | Yes |
| 231 | PCSK1N |  |  | 3332 | -0.981 | -0.2391 | Yes |
| 232 | MYBL2 |  |  | 3340 | -1.006 | -0.2356 | Yes |
| 233 | DEK |  |  | 3344 | -1.025 | -0.2308 | Yes |
| 234 | RAD51AP1 |  |  | 3345 | -1.030 | -0.2250 | Yes |
| 235 | PBK |  |  | 3354 | -1.046 | -0.2215 | Yes |
| 236 | HIST2H2AC |  |  | 3363 | -1.077 | -0.2179 | Yes |
| 237 | ESCO2 |  |  | 3367 | -1.088 | -0.2127 | Yes |
| 238 | NCAPG |  |  | 3370 | -1.098 | -0.2072 | Yes |
| 239 | MCM7 |  |  | 3384 | -1.147 | -0.2047 | Yes |
| 240 | GINS2 |  |  | 3386 | -1.156 | -0.1985 | Yes |
| 241 | DHFR |  |  | 3387 | -1.159 | -0.1920 | Yes |
| 242 | HMGB2 |  |  | 3390 | -1.184 | -0.1859 | Yes |
| 243 | CLSPN |  |  | 3391 | -1.184 | -0.1793 | Yes |
| 244 | UBE2S |  |  | 3396 | -1.205 | -0.1737 | Yes |
| 245 | CENPU |  |  | 3398 | -1.230 | -0.1671 | Yes |
| 246 | HIST1H1E |  |  | 3401 | -1.237 | -0.1608 | Yes |
| 247 | IL11 |  |  | 3403 | -1.239 | -0.1541 | Yes |
| 248 | LYPD1 |  |  | 3412 | -1.270 | -0.1494 | Yes |
| 249 | ATAD2 |  |  | 3415 | -1.278 | -0.1428 | Yes |
| 250 | RRM2 |  |  | 3420 | -1.297 | -0.1367 | Yes |
| 251 | CLU |  |  | 3424 | -1.329 | -0.1302 | Yes |
| 252 | HIST1H1C |  |  | 3427 | -1.335 | -0.1233 | Yes |
| 253 | IGFBP3 |  |  | 3433 | -1.384 | -0.1170 | Yes |
| 254 | TYMS |  |  | 3436 | -1.391 | -0.1098 | Yes |
| 255 | CDH2 |  |  | 3439 | -1.439 | -0.1023 | Yes |
| 256 | AKAP12 |  |  | 3444 | -1.501 | -0.0951 | Yes |
| 257 | HIST1H1D |  |  | 3466 | -1.743 | -0.0918 | Yes |
| 258 | HIST1H1B |  |  | 3467 | -1.770 | -0.0818 | Yes |
| 259 | GPM6B |  |  | 3478 | -1.861 | -0.0744 | Yes |
| 260 | SCRG1 |  |  | 3480 | -1.876 | -0.0642 | Yes |
| 261 | S100B |  |  | 3489 | -2.109 | -0.0548 | Yes |
| 262 | BEX1 |  |  | 3490 | -2.151 | -0.0426 | Yes |
| 263 | HIST1H4C |  |  | 3493 | -2.248 | -0.0306 | Yes |
| 264 | FABP7 |  |  | 3496 | -2.308 | -0.0182 | Yes |
| 265 | PTPRZ1 |  |  | 3510 | -4.002 | 0.0003 | Yes |
Table: GSEA details [plain text format]

  

Fig 2: FELDMAN\_2020\_S-G2: Random ES distribution      
 Gene set null distribution of ES for **FELDMAN\_2020\_S-G2**
